# Case report: Aggressive progression of acute heart failure due to juvenile tuberculosis-associated Takayasu arteritis with aortic stenosis and thrombosis

**DOI:** 10.3389/fcvm.2023.1076118

**Published:** 2023-03-21

**Authors:** Wenjie Xuan, Zhaoling Wang, Jinjing Lin, Lixia Zou, Xisheng Xu, Xinghui Yang, Yiping Xu, Yan Zhang, Qi Zheng, Xuefeng Xu, Meiping Lu

**Affiliations:** ^1^ Department of Rheumatology Immunology and Allergy, Children's Hospital of Zhejiang University School of Medicine, National Clinical Research Center for Child Health, Hangzhou, China; ^2^ Department of Pediatrics, Shaoxing People's Hospital (Shaoxing Hospital, Zhejiang University School of Medicine), Shaoxing, China; ^3^ Department of Radiology, Children's Hospital of Zhejiang University School of Medicine, National Clinical Research Center for Child Health, Hangzhou, China

**Keywords:** juvenile, Takayasu arteritis, acute heart failure, pulmonary hypertension, thrombosis, surgery

## Abstract

**Background:**

Takayasu arteritis (TA) is a chronic granulomatous vasculitis with unknown pathophysiology. TA with severe aortic obstruction has a poor prognosis. However, the efficacy of biologics and appropriate timing of surgical intervention remain controversial. We report a case of tuberculosis (TB)-associated TA with aggressive acute heart failure (AHF), pulmonary hypertension (PH), thrombosis, and seizure, who failed to survive after surgery.

**Case presentation:**

A 10-year-old boy who developed a cough with chest tightness, shortness of breath, hemoptysis with reduced left ventricular ejection fraction, PH, and increased C-reactive protein and erythrocyte sedimentation rate was hospitalized at the pediatric intensive care unit of our hospital. He had strongly positive purified protein derivative skin test and interferon-gamma release assay result. Computed tomography angiography (CTA) showed occlusion of proximal left subclavian artery and stenosis of descending aorta and upper abdominal aorta. His condition did not improve after administration of milrinone, diuretics, antihypertensive agents, and intravenous methylprednisolone pulse followed by oral prednisone. Intravenous tocilizumab was administered for five doses, followed by two doses of infliximab, but his HF worsened, and CTA on day 77 showed complete occlusion of the descending aorta with large thrombus. He had a seizure on day 99 with deterioration of renal function. Balloon angioplasty and catheter-directed thrombolysis were performed on day 127. Unfortunately, the child's heart function continued to deteriorate and died on day 133.

**Conclusion:**

TB infection may be related to juvenile TA. The biologics, thrombolysis, and surgical intervention failed to achieve the anticipated effect in our case with aggressive AHF due to severe aortic stenosis and thrombosis. More studies are needed to determine the role of biologics and surgery in such dire cases.

## Introduction

Takayasu arteritis (TA) is a chronic, granulomatous vasculitis that predominantly involves the aorta and its main branches (1). TA may lead to fatal complications, such as heart failure (HF), myocardial infarction, dissecting aneurysm, and stroke (2). Juvenile TA is less common and has an insidious onset. The pathogenesis of TA may be related to infection, genetic susceptibility, and immune abnormalities. Tuberculosis (TB) was one of the leading causes of death from a single infectious pathogen, with an estimated 2 million TB deaths in 2019 in the world (3). The prevalence of TB was relatively high in Southeast Asia (44%), Africa (25%), and Western Pacific (18%) (3). Studies have shown that TA is closely associated with TB infection in adults, of which 9.9% had TB (4). Pedreira and Santiago (5) reported that active or latent TB accounted for 6.3%–20% or 20%–82% of patients with TA, respectively. However, there was no difference in clinical presentation in TA with or without TB infection (6). TB-associated juvenile TA is rare (7, 8). Treatment of juvenile TA is based on the approaches used for adults, but the rapid onset and severity of the disease in children means that treatment options are very limited, and the efficacy of biologics or surgical interventions is controversial (9, 10). Herein, we reported an aggressive case of TB-associated TA in a Chinese boy, who presented with refractory acute heart failure (AHF), pulmonary hypertension (PH), and seizure due to severe aortic stenosis and associated thrombosis. Balloon angioplasty and catheter-directed thrombolysis were attempted, but unfortunately, those did not prevent his demise on day 133.

## Case presentation

A 10-year-old Chinese boy was transferred to the pediatric intensive care unit (PICU) of our hospital with AHF, PH, and hypertension. Ten months prior to this admission, he presented to the local healthcare providers with “chest pain,” increased erythrocyte sedimentation rate (ESR: 39–52 mm/h), and C-reactive protein (CRP: 22.6–29.1 mg/L), and chest computed tomography (CT) scan demonstrated calcified left hilar lymph nodes. Chest pain improved after antibiotic therapy for 1 week (no detail regarding the medications).

On this admission, he returned to the local hospital with a 2-week history of cough, chest tightness, and shortness of breath. He was diagnosed with AHF, PH, and hypertension and transferred to our hospital. The patient had received routine vaccinations including Bacillus Calmette-Guerin (BCG). His growth and development were normal.

**Figure 1:**
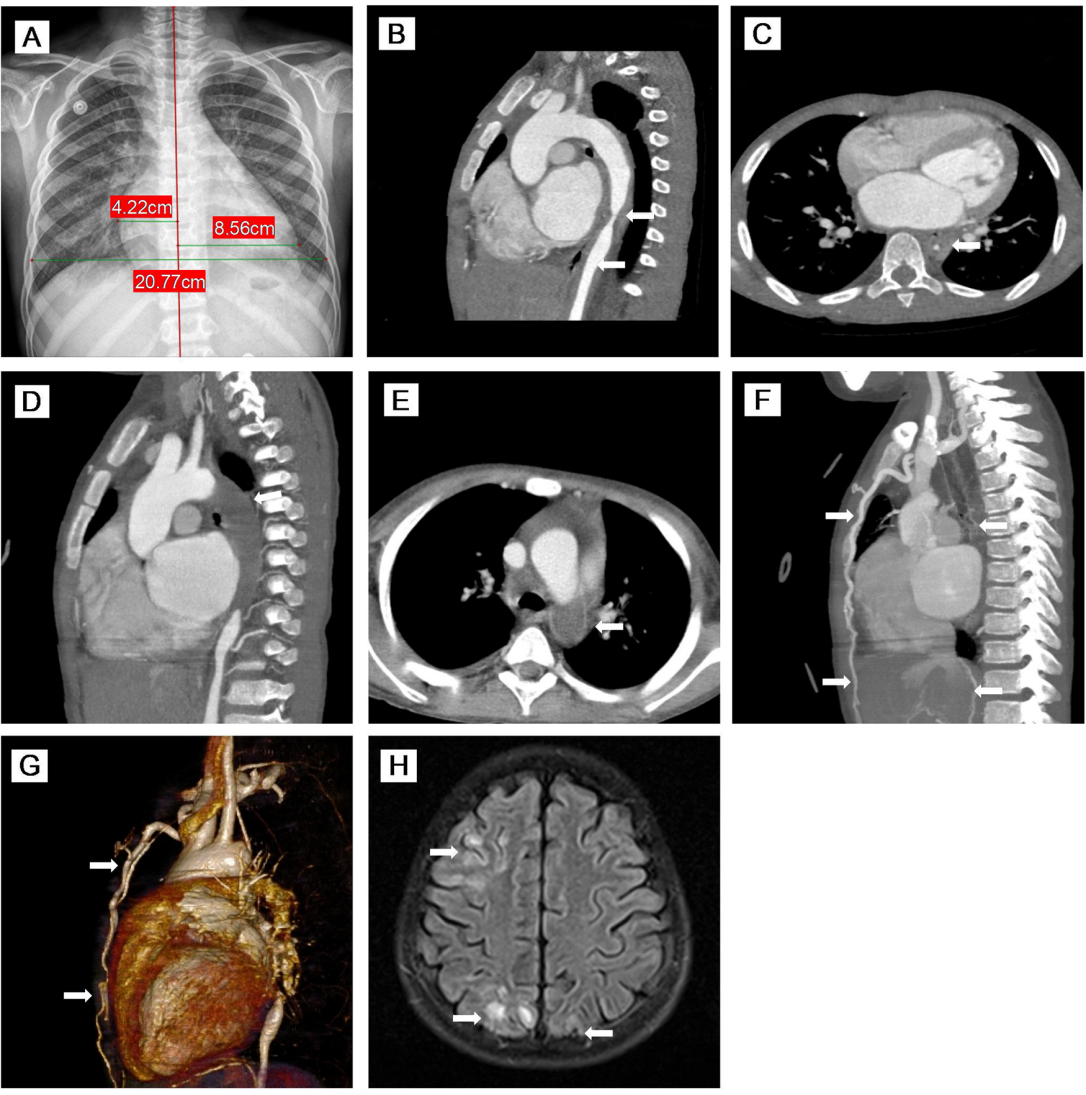
(**A**) Chest x-ray showed significant cardiomegaly. (**B–E**) MPR images of thoracic CTA. (**B**) Sagittal view of CTA demonstrated stenosis of the descending aorta and upper abdominal aorta with thickening of the vascular wall. (**C**) Axial view of CTA showed that the descending aorta was stenosed to 3.1 mm. (**D,E**) Sagittal oblique view and axial view of CTA indicated complete occlusion of the descending aorta with large thrombus (76 mm in length). (**F,G**) MIP image and VR reconstruction of CTA revealed complete occlusion of the descending aorta with large thrombus (76 mm in length) and extensive collateral circulation (twisted and dilated right intrathoracic artery, numerous open collateral vessels in the neck and upper mediastinum). (**H**) Axial view of brain MR imaging revealed high signals on T2 FLAIR images of cortical and subcortical areas of both the occipital lobes and right frontal-parietal lobe. MPR, multiplanar reconstruction; CTA, computed tomography angiography; MIP, maximum intensity projection; VR, volume rendered; MR, magnetic resonance; FLAIR, fluid-attenuation inversion recovery.

Physical examination on admission showed the following: heart rate 120/min, respiratory rate 26/min, and blood pressure (BP) 159/103 mmHg in his right arm, 124/93 mmHg in his left arm, 87/55 mmHg in his right leg, and 121/106 mmHg in the left leg. Distended jugular veins and weak left radial artery pulsation were found. Class IV systolic murmur in the precordial region was heard. There was no enlargement of liver and spleen. Laboratory evaluations revealed increased N-terminal pro-brain natriuretic peptide (NT-proBNP: 19052 pg/mL), CRP (8.45 mg/L), ESR (32 mm/h), serum creatinine (86 μmol/L), and urea (15.68 mmol/L) with decreased blood potassium (2.9 mmol/L), sodium (128 mmo1/L), and hemoglobin (84 g/L). The purified protein derivative (PPD) skin test and interferon-gamma release assay (IGRA) result were strongly positive. However, all microbiological analyses including TB culture and pathogenic microorganism sequencing were negative. Serum immunoglobulin and complements (C3 and C4) were normal. Antinuclear antibody (ANA) and antineutrophil cytoplasmic antibody (ANCA) were negative. Echocardiography revealed reduced left ventricular ejection fraction (LVEF: 40%) with left heart dilatation, PH (75 mmHg), and moderate mitral insufficiency. Chest x-ray showed significant cardiomegaly (Figure 1A). Chest CT showed calcified left hilar lymph nodes, enlarged mediastinal lymph nodes, patchy and slightly high-density shadows in both lungs, and a small amount of bilateral pleural effusion. CT angiography (CTA) showed occlusion of the proximal left subclavian artery and stenosis of the lumen of the descending and upper abdominal aorta with extensive thickening of the vascular wall (Figures 1B,C). Whole exome sequencing did not indicate any abnormal mutations. Based on the radiological findings and laboratory examinations, he was diagnosed as TB-associated TA (11) complicated with AHF and PH.

**Figure 2:**
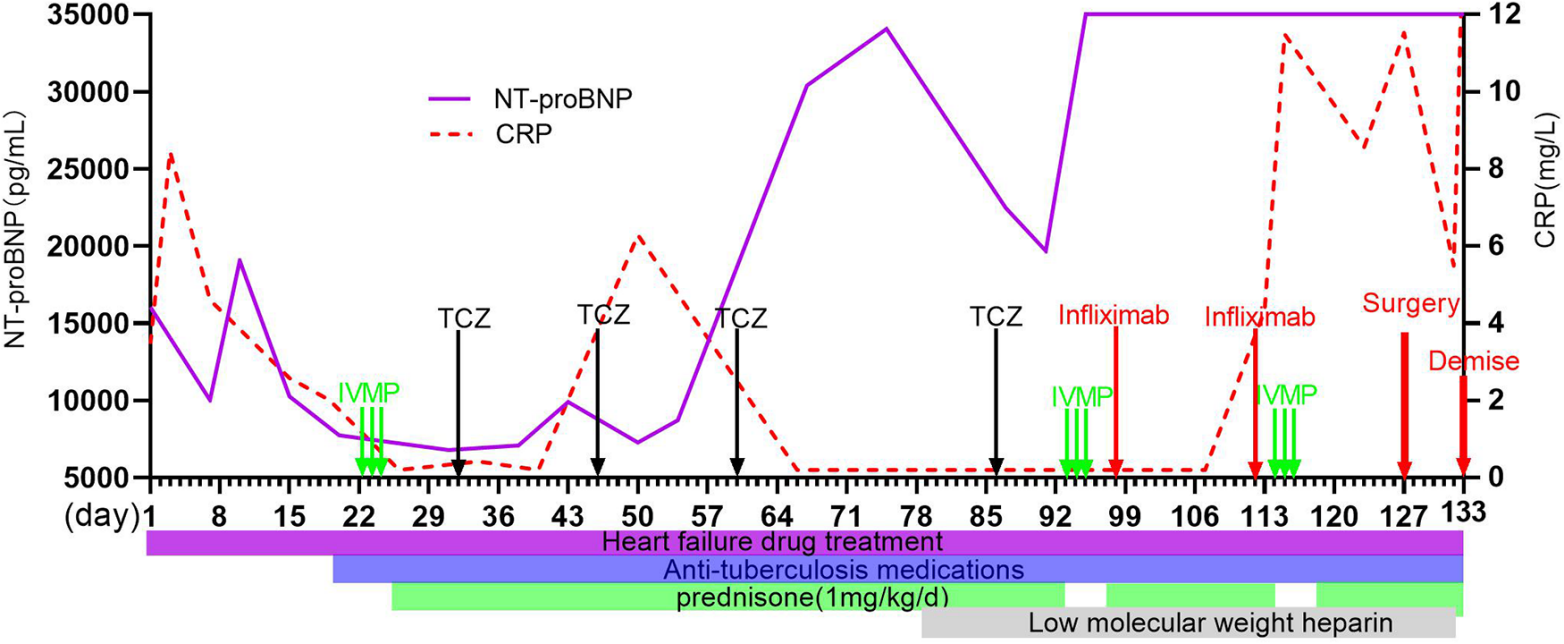
Clinical course of this case and sequential changes in NT-proBNP and CRP. NT-proBNP, N-terminal pro-brain natriuretic peptide; CRP, C-reactive protein; IVMP, intravascular methylprednisolone pulse; TCZ, tocilizumab.

The boy was initially treated with bed rest, oxygen inhalation, milrinone, diuretics, captopril, bosentan, and antibiotics including anti-TB medications. He developed hemoptysis on day 8. Intravenous methylprednisolone pulse (IVMP) therapy (10 mg/kg/day) was given on days 22–24, followed by 1 mg/kg of daily oral prednisone. Metoprolol was added on day 28. However, subxiphoid and right upper abdominal pain was recurrent during hospitalization and worsened on day 29. AHF, PH, BP, heart rate, cardiac function, chest tightness, and shortness of breath did not improve notably. On day 32, intravenous tocilizumab (TCZ) was initiated at a dose of 8 mg/kg following the 0-, 2-, and 4-week regimen (Figure 2). Coughing and hemoptysis resolved. Chest tightness, shortness of breath, abdominal pain, BP (115/82 mmHg on the right arm) and heart rate (102 beats/min), LVEF (45%), and PH (47 mmHg) were slightly improved after three doses of TCZ. He was discharged on day 60.

The child was re-admitted on day 66 due to worsening HF with lower LVEF (30%), higher PH (70 mmHg), and normal CRP and ESR. During this admission, his BP fluctuated from 175/105–123/74 mmHg, and hemoptysis recurred. Repeated CTA on day 77 and day 92 showed complete occlusion of the descending aorta with large thrombus (76 mm in length) (Figures 1D,E) and extensive collateral circulation (twisted and dilated right intrathoracic artery, numerous open collateral vessels in the neck and upper mediastinum) (Figures 1F,G). In addition to the same medications as the first admission, subcutaneous low-molecular-weight heparin was started on day 78. IVMP therapy (10 mg/kg/day) was administered again on days 93–95.

The patient developed seizure and loss of consciousness while afebrile on day 99. Seizure was controlled with intravenous diazepam. Cranial magnetic resonance angiography (MRA) showed narrowed lumen of the anterior, middle, and posterior cerebral arteries. Brain MR imaging revealed strong signals on T2 fluid-attenuation inversion recovery (FLAIR) images of the cortical and subcortical areas of both the occipital lobes and right frontal-parietal lobe (Figure 1H). After failing to respond to another two doses of TCZ, intravenous infliximab was given for two doses on day 98 and day 112, and IVMP therapy (10 mg/kg/day) again on days 114–116. However, there were still no signs of improvement, and his renal function worsened. Given the complete occlusion of the aorta with large thrombus and aggravating life-threatening HF without response to any medication, balloon angioplasty was performed and catheter-directed thrombolysis with urokinase and heparin was started on day 127. Four days after surgery, the patient worsened suddenly with increased shortness of breath, decreased heart rate, and dropping of oxygen saturation to 60%. The patient died on day 133 (Figure 2).

## Discussion and conclusion

In our case, the boy presented with hypertension and differential limb systolic pressure of >10 mmHg, increased ESR of 32 mm/h, and CRP of 8.45 mg/L. CTA showed the involvement of the left subclavian artery, descending aorta, and abdominal aorta. All microbiological analyses in our case were negative except for the positive PPD skin test and IGRA result. Treatment with antibiotics including anti-TB medications was ineffective. There was no evidence of other immune inflammatory diseases such as systemic lupus erythematosus and ANCA-associated vasculitis based on the clinical and laboratory features. TA was diagnosed according to the diagnostic criteria developed by the European League Against Rheumatism (EULAR)/Paediatric Rheumatology International Trials Organisation/Paediatric Rheumatology European Society (11). The clinical course was complicated with refractory HF, PH, thrombosis, and central nervous system involvement and renal dysfunction.

HF is the leading cause of death in adult TA patients (12). Zhang et al. (13) analyzed 163 adult patients with TA, including 61 cases with HF. During a median follow-up period of 887 days, all 11 fatal cases were in the HF group. AHF is less common but more malignant than chronic HF in TA patients. Fan et al. (14) reported five children with TA complicated with AHF, of which two patients had improved, two died, and one was lost to follow-up. Our case with AHF due to severe stenosis of descending aorta failed to all treatments and succumbed to his disease.

PH is another serious complication of TA, and the incidence rate is approximately 0%–17.8%. PH occurs because of (i) precapillary causes, such as pulmonary disease, chronic hypoxia, and chronic thromboembolic disease, and (II) postcapillary causes, such as left heart disease, aortic regurgitation, or disease associated with pulmonary arteritis (15, 16). PH is mostly asymptomatic clinically, while some patients may present with dyspnea, decreased activity tolerance, low oxygen saturation, occasional pulmonary hemorrhage, and, in severe cases, hemoptysis and HF (15). In our case, he presented with cough, chest tightness, shortness of breath, hemoptysis with persistent lower LVEF, and high NT-proBNP but without pulmonary artery involvement in CTA. It indicated that the refractory PH and hemoptysis may be secondary to left heart failure/dysfunction as a result of severe stenosis or complete occlusion of the descending aorta.

The patient developed seizure with abnormal cranial MRA and T2 FLAIR images on day 99. Seizures may be caused by stroke, hypertensive encephalopathy, posterior reversible encephalopathy syndrome (PRES), and other reasons. Neurological symptoms in TA are usually associated with decreased blood flow due to steno-occlusive lesions and/or shifting of the blood flow (steal) (17, 18). PRES is extremely rare in TA. The mechanism of PRES is not fully understood but may be related to the blood–brain barrier disruption with fluid transudation caused by high BP (19). In our case, the patient received long-term intravenous methylprednisolone and several IVMP and suffered from persistent hypertension. Seizures may be related to both hypertension induced PRES and narrowed lumen of arteries according to imaging findings.

The EULAR recommendations for the management of TA included glucocorticoids and conventional synthetic disease-modifying antirheumatic drugs, followed by TCZ or TNF-inhibitors (20). In our case, TCZ at the first hospitalization led to a slight transient improvement. However, his HF worsened with increased aortic stenosis and complete occlusion of the descending aorta despite five doses of TCZ. Changing TCZ to a TNF-inhibitor (infliximab) also failed to reverse the condition. Our case indicated that efficacy of biologics was poor in TA with severe stenosis or occlusion of aorta induced HF. As aortic obstruction may contribute to aggressive HF, which could result in death from cardiopulmonary failure, a desperate final attempt to reverse the clinical course was made with surgical intervention.

Previous studies revealed that surgery was helpful to relieve vascular obstruction in patients with severe ischemia of vital organs and limbs, intractable hypertension, and refractory HF (20, 21). Although in most cases surgery is beneficial, it is always accompanied by risks (Table 1). Two of five cases of juvenile TA complicated with HF survived with surgery (14). An 8-year-old patient with TA who underwent surgical treatment for the complication of aortic occlusion and HF was stable with 10 years of follow-up (22). Nakata et al. (9) reported that a 1-year-old girl with TA-induced long-segment occlusion of the distal thoracic aorta developed cardiogenic shock but survived after surgery. However, another case of a 3.5-month-old boy with TA died after surgery (10). In general, surgical intervention should not be performed until TA patients reach an inactive state with normal ESR, CRP, and stable imaging (23). In most TA patients, increased ESR and CRP correlated with active disease status. However, about 20% of TA patients have normal laboratory parameters despite having active disease (24). There was evidence that the disease activity significantly might increase the risks of anastomotic dehiscence or restenosis (25). This issue makes it difficult for the clinicians to determine the optimal time in surgical intervention. In our case, given the progressive HF and associated decline in renal function, balloon angioplasty and catheter-directed thrombolysis with urokinase and heparin were done in a desperate final attempt to save his life. Unfortunately, the boy died 6 days after surgery. This case suggested that TA with severe HF due to severe aortic stenosis and occlusion had very poor prognosis. The optimal time for surgical intervention remains problematic.

**Table 1:** Review of surgical modalities and outcomes in juvenile Takayasu arteritis involving aorta.

First author, year (reference)	Sex	Age	Heart failure	Involved vessels	Surgical modalities	Outcome	Follow-up
Fan et al., 2018 (14)	F	Adolescent	Yes	Stenosis of distal thoracic aorta	Stent implantation	Improvement	3 years
Fan et al., 2018 (14)	F	Adolescent	Yes	Stenosis from thoracic to abdominal aorta	Balloon angioplasty	Improvement	6 months
Sugawara et al., 2020 (22)	F	8 years	Yes	Occlusion of descending aorta	Axillo-external iliac artery bypass	Improvement	10 years
Nakata et al., 2022 (9)	F	1 year	Yes	Occlusion of distal thoracic aorta	Graft interpose	Improvement	2 months
Pavic et al., 2019 (10)	M	3.5 months	Yes	Thrombosis of distal aorta and iliac arteries	Thrombectomy	Death	—

F, female; M, male.

In summary, TB infection may be related to juvenile TA. It is a must to investigate early in all TA, even if there are no signs or symptoms, especially in TB-endemic countries. TA patients with aggressive AHF due to severe aortic stenosis and thrombosis have a poor prognosis, and biologics are of limited efficacy. Our case pointed out that timing of surgical intervention with balloon angioplasty and catheter-directed thrombolysis may be critical in disease outcome, but it is extremely difficult to pinpoint when the optimal time may be. More studies are needed to better address this issue.

## Data Availability

The original contributions presented in the study are included in the article/Supplementary Material, further inquiries can be directed to the corresponding author.
